# Development of siRNA therapeutics to combat microbial infections: a bibliometric analysis

**DOI:** 10.3389/fcimb.2025.1697880

**Published:** 2025-10-24

**Authors:** Yi He, Yulin Yuan, Meihua Zhou, Miao Li, Lingjin Li, Chunhong Li, Xiaocui Liang, Panyan Liu, Wei Wang, Zhenfeng Deng

**Affiliations:** ^1^ Infection Diagnosis Center, Guangxi KingMed Diagnostics, Nanning, Guangxi, China; ^2^ Department of Clinical Laboratory, the People’s Hospital of Guangxi Zhuang Autonomous Region, Nanning, Guangxi, China; ^3^ Department of Intensive Care Unit, the People’s Hospital of Guangxi Zhuang Autonomous Region, Nanning, Guangxi, China

**Keywords:** siRNA therapeutics, microbial infections, small interfering RNA, RNA interference, delivery system, COVID-19, bibliometric analysis

## Abstract

**Background:**

The rise of antimicrobial resistance and the COVID-19 pandemic highlight the limitations of traditional therapies. Small interfering RNA (siRNA) therapeutics, which utilize RNA interference for targeted gene silencing, present a promising approach to combating microbial infections. However, research in this area remains fragmented. This study employs a comprehensive bibliometric analysis to chart research trends and inform future directions.

**Methods:**

A total of 8,426 publications from the Web of Science Core Collection (2001–2025) were analyzed using CiteSpace and VOSviewer software to examine annual publication trends, geographic and institutional contributions, author networks, journal impacts, and keyword evolution. Data extraction focused on English-language articles.

**Results:**

The publication trends for siRNA therapeutics in microbial infections have evolved in three phases: rapid growth, stabilization at a peak, and subsequent cyclical fluctuations. Research contributions spanned 99 countries and regions, with 5,564 institutions and 1,234 journals involved. China (2,849 publications) and the United States (2,820 publications) led in publication volume. While the United States maintained dominance in academic influence and collaboration, China has steadily increased its research output in this area. The Journal of Virology emerged as the leading journal in terms of both productivity and citation impact. Key research areas include delivery systems, target selection, manufacturing technologies, antiviral therapeutics, and combination therapies. The field has shifted from basic mechanistic studies to clinical applications, with future research poised to focus on organ-specific delivery beyond the liver, exploration of diverse administration routes, integration of artificial intelligence-driven strategies, and enhanced global collaboration.

**Conclusion:**

This bibliometric analysis offers a comprehensive overview of siRNA therapeutics for microbial infections, highlighting collaboration networks and academic influence across authors, countries, institutions, and journals. The study provides valuable insights into current research trends and serves as a foundational reference for guiding future collaborative efforts and innovations in this field.

## 1 Introduction

The emergence of antimicrobial resistance and the COVID-19 pandemic has exposed significant vulnerabilities in conventional therapeutic strategies. Traditional antibiotics and antivirals, which target conserved microbial structures or enzymes, are increasingly ineffective due to pathogen evolution and mutation, and exhibit limited efficacy against viruses (Cook and Wright, 2022; Karim et al., 2023). Rapidly mutating viruses such as severe acute respiratory syndrome coronavirus 2 (SARS-CoV-2), persistent viruses like hepatitis B virus (HBV), and pathogens such as methicillin-resistant *Staphylococcus aureus* (MRSA) exemplify the constraints of existing therapies. These challenges demand innovative approaches that adapt to genetic variability and overcome resistance mechanisms. In this context, small interfering RNA (siRNA) has emerged as a transformative modality, offering a promising strategy to combat microbial infections through sequence-specific gene silencing that disrupts microbial replication, virulence, or host dependency factors (Ebenezer et al., 2025). Unlike small-molecule drugs, siRNA enables precise targeting of pathogen genomes or host pathways, minimizing off-target effects and mitigating the risk of drug resistance. Early successes, such as siRNA-mediated suppression of HBV surface antigen (HBsAg) in clinical trials (Yuen et al., 2020), demonstrate its potential to redefine antimicrobial therapeutics.

SiRNA therapeutics operate through RNA interference (RNAi), a conserved mechanism that facilitates the degradation of complementary messenger RNA (mRNA). The process begins when siRNA duplexes are incorporated into the RNA-induced silencing complex (RISC), where the guide strand directs target recognition and cleavage (Carthew and Sontheimer, 2009). The specificity of siRNA is derived from its ability to bind to unique genomic sequences, making it particularly effective against pathogens with high mutation rates. For instance, siRNA targeting ultra-conserved regions of SARS-CoV-2 demonstrated broad-spectrum activity across variants, highlighting its adaptability (Xu et al., 2022; Rabdano et al., 2024). Moreover, chemical modifications and advanced delivery systems, such as lipid nanoparticles (LNPs) and triantennary N-acetylgalactosamine (tri-GalNAc) conjugates, have improved siRNA stability, biodistribution, and cellular uptake (Hu et al., 2020). These innovations address early challenges of immunogenicity and inefficient delivery, facilitating clinical applications for infections like HBV and human immunodeficiency virus (HIV).

Despite these advancements, significant challenges remain in the application of siRNA for microbial infections. Bacterial and fungal targets are underrepresented due to the absence of RNAi machinery in prokaryotes and delivery barriers, while viral applications dominate the field (Ebenezer et al., 2025). Additionally, clinical heterogeneity in outcomes, stemming from variability in delivery platforms and dosing regimens, highlights the need for standardized methodologies. Bibliometric analyses reveal fragmented research efforts, with notable disparities in geographic contributions and thematic focus. A comprehensive evaluation of the field’s intellectual structure and emerging trends is therefore crucial to prioritize high-impact research, foster global collaboration, and address translational gaps. This study aims to construct a visualization model to assess current research trends, track the evolution of the field, and forecast future directions. By providing an extensive visual knowledge map, it offers critical insights for researchers and guides future initiatives in siRNA therapeutics for microbial infections.

## 2 Methods

### 2.1 Database and search strategy

**Figure 1:**
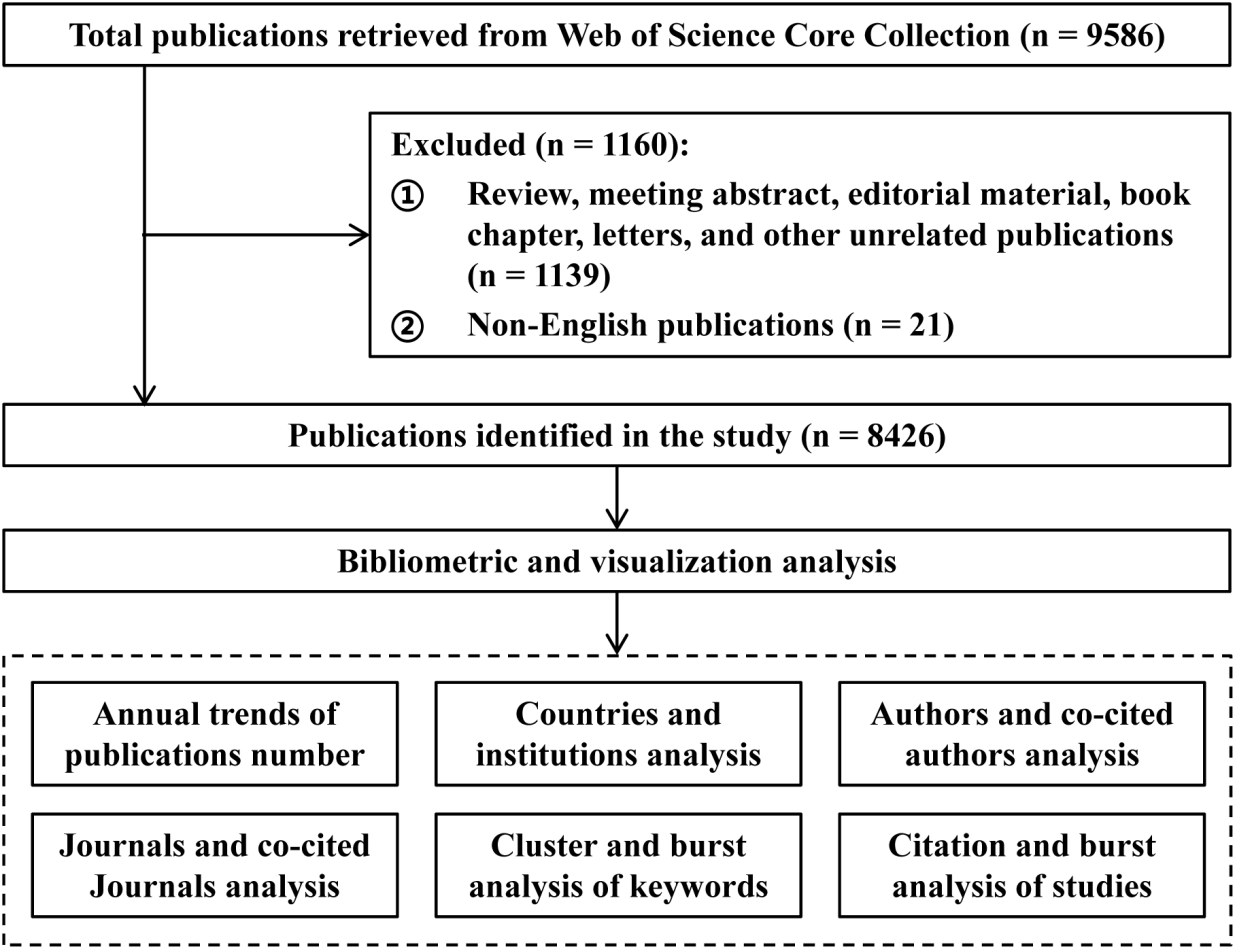
Flow diagram of the study.

All publications in this study were sourced from the Web of Science Core Collection (WOSCC), a database indexing over 12,000 high-impact academic journals. The retrieval strategy employed the following search terms: TS = (“small interfering RNA” OR “siRNA”) AND TS = (infection OR infectious diseases OR pathogens OR viruses OR bacteria OR fungi OR parasites) NOT TS = (plant OR agriculture OR agronomy OR environment OR ecology OR food OR veterinary OR entomology). To focus on human infections, the search excluded clearly irrelevant fields such as agriculture, environmental sciences, food science, and veterinary medicine using the “NOT” operator. The search period covered the database’s inception through June 30, 2025, with the language limited to English. To ensure that the included literature is original research that has undergone strict peer review and to maintain the comparability of influence among different studies, the document types for this study is limited to articles only, avoiding other types of literature such as reviews and conference abstracts that may interfere with the analysis results. The literature screening process for this study is illustrated in **Figure 1**.

### 2.2 Data analysis

Data, including title, authors, publication year, country/region, institution, keywords, citations, abstracts, and references, were extracted from the WOSCC database in plain text and tab-delimited file formats. The impact factor from the Journal Citation Reports (JCR) 2024 was utilized for analysis. Two authors independently screened the literature based on predefined inclusion and exclusion criteria, with a focus on English-language articles. For bibliometric analysis, VOSviewer 1.6.19 (Leiden University, Netherlands) was used to extract qualifying data and perform visual analyses on country/region, institution, author, keyword, and study trends. Data cleansing was performed using VOSviewer’s built-in tools to systematically remove duplicate, irrelevant, or invalid entries. Concurrently, manual verification was conducted on entries containing missing information and ambiguous abbreviations (such as author names, institutional affiliations, and journal titles) to ensure the accuracy of the visualization results. CiteSpace 6.1.R6 (Drexel University, Pennsylvania, USA) was used for burst analysis of keywords and studies. Visualization, including line graphs, geographical distribution maps, chord diagrams, and stacked area graphs, was implemented using the CNSKonwall platform (https://cnsknowall.com/).

## 3 Results

### 3.1 Annual trends in the publications number

**Figure 2:**
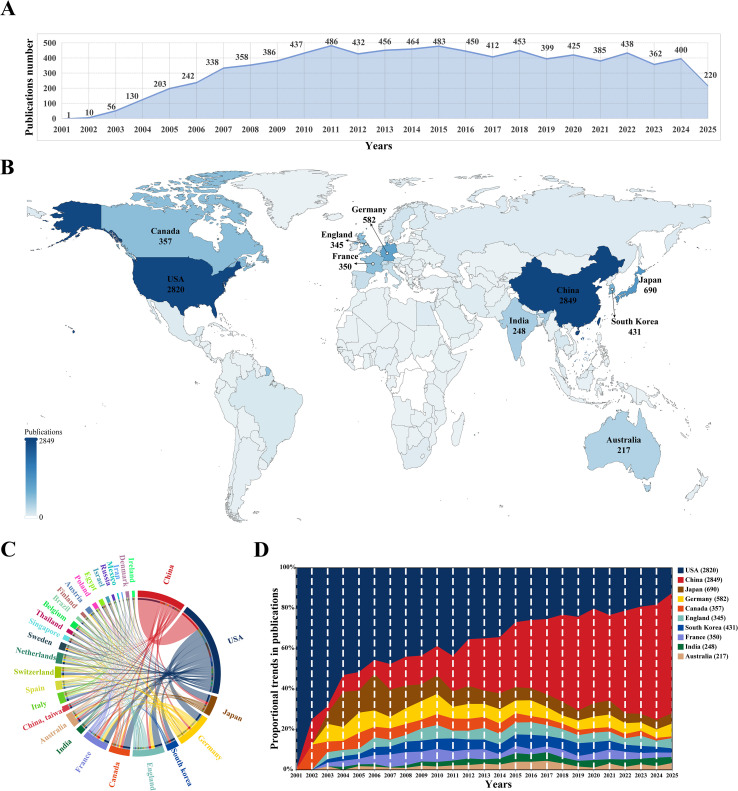
**(A)** Annual trends in publication numbers. **(B)** Geographical distribution of countries/regions in global publications. Darker colors indicate a higher number of publications in the field of siRNA therapeutics for microbial infections, with the top 10 highly productive countries highlighted. **(C)** Co-occurrence map of countries/regions in this research field. The arc length on the circumference is proportional to publication numbers, while thicker connecting lines indicate stronger collaborative relationships. **(D)** Stacked area graph of publication proportion trends in the top 10 highly productive countries over time.

Through comprehensive manual screening, a total of 8,426 articles on siRNA therapeutics for microbial infections were identified from 2001 to 2025. As shown in **Figure 2A**, the annual publication trends exhibit a three-phase pattern of “explosive growth, double-peak stable development, and fluctuating adjustments.” During the early phase (2001–2009), the number of publications experienced rapid growth, with an average annual growth rate of 110.53%, increasing from 1 to 386 articles per year. In the middle phase (2010–2018), growth slowed to an average annual rate of 0.45%, with peaks of 486 publications in 2011 and 483 in 2015. The most recent phase (2019–present) entered a period of cyclical fluctuations, with alternating increases and decreases in annual publications, ultimately stabilizing at approximately 400 publications by 2024. Notably, the 2025 data only includes records up to June 30 (220 publications) and remains incomplete. Overall, the field has transitioned from rapid early growth to a phase of periodic adjustments, maintaining an average annual growth rate of 29.76% from 2001 to 2024.

### 3.2 Distribution of countries/regions and institutions

The analysis revealed contributions from 5,564 institutions across 99 countries/regions to the field of siRNA therapeutics for microbial infections. **Figure 2B** shows the geographical distribution of these countries/regions in global publications. **Table 1** lists the top 10 most productive countries, including their total citations (TC), average citations, and total link strength (TLS). China (2,849) and the United States (2,820) lead in publication volume, ranking first and second, respectively, followed by Japan (690), Germany (582), and South Korea (431). However, the USA leads in TC, significantly surpassing China, Japan, Germany, and Canada, which occupy the second to fifth positions. The TLS, which reflects the intensity of research collaboration, shows that the USA and several European countries, particularly Germany, France, and the UK, lead in academic cooperation networks, occupying four of the top five TLS positions, with China ranking second. **Figure 2C** illustrates the co-occurrence map among countries/regions involved in this research topic, while **Figure 2D** presents the proportional trends in publication numbers for the top 10 countries over different years, shown through a stacked area graph.

**Table 1:** Top 10 highly productive countries.

Rank	Country	PN	TC	AC	TLS
1	China	2849	63710	22.36	716
2	USA	2820	166397	59.01	1471
3	Japan	690	29702	43.05	310
4	Germany	582	25898	44.50	575
5	South Korea	431	13736	31.87	185
6	Canada	357	20206	56.60	306
7	France	350	19449	55.57	417
8	England	345	16275	47.17	462
9	India	248	5420	21.85	120
10	Australia	217	7593	34.99	222

PN, publications number; TC, total citations; AC, average citations; TLS, total link strength.

**Figure 3:**
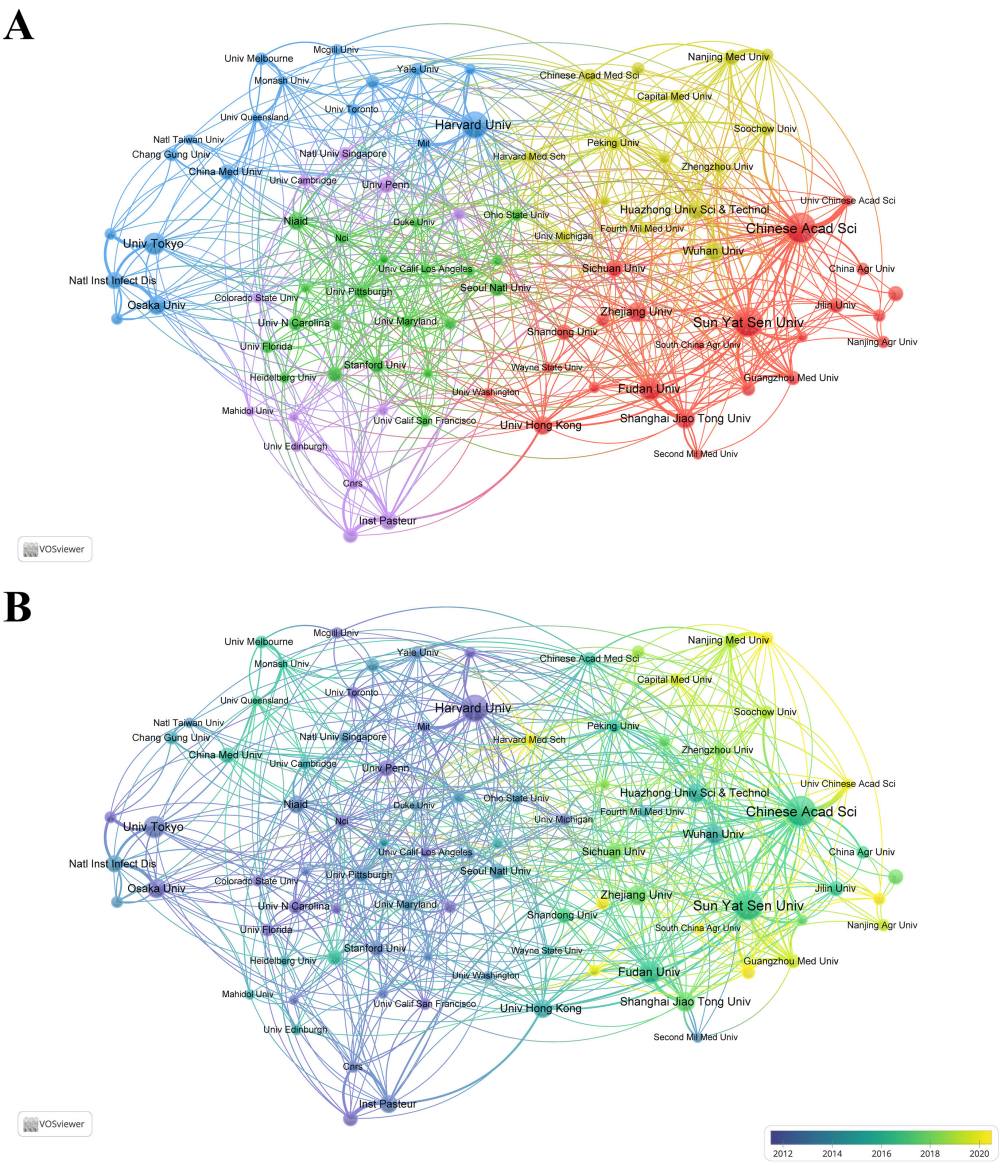
**(A)** Collaborative network visualization and cluster analysis of institutions. **(B)** Visualization of overlay time evolution for institutions.

**Table 2** lists the top 10 most productive institutions, with 7 from China, 2 from Japan, and 1 from the USA. The Chinese Academy of Sciences leads with 141 publications, while Harvard University ranks first in TC with 13,816 citations. Both institutions tie for first place in TLS. **Figure 3A** shows a cluster analysis of institutions (with a minimum of 30 publications), revealing five closely collaborating clusters, each distinguished by different colors. This clustering highlights the collaborative networks among institutions, providing insights for future inter-institutional cooperation. **Figure 3B** further illustrates the evolution of these institutions over time. Additionally, the top 10 institutions in terms of TC are listed in **Supplementary Table S1**, with Harvard University, the University of Pennsylvania, and the Massachusetts Institute of Technology (MIT) ranking the top three, all from the USA, reflecting their strong academic influence and significant contributions to this field of research.

**Table 2:** Top 10 highly productive institutions.

Rank	Institution	Country	PN	TC	AC	TLS
1	Chinese Acad Sci	China	141	4759	33.75	229
2	Sun Yat Sen Univ	China	136	3137	23.07	132
3	Harvard Univ	USA	122	13816	113.25	229
4	Fudan Univ	China	99	2711	27.38	110
5	Univ Tokyo	Japan	95	4516	47.54	148
6	Shanghai Jiao Tong Univ	China	85	1722	20.26	102
7	Zhejiang Univ	China	85	2887	33.96	75
8	Huazhong Univ Sci & Technol	China	82	2243	27.35	68
9	Wuhan Univ	China	82	2511	30.62	71
10	Osaka Univ	Japan	80	5476	68.45	114

PN, publications number; TC, total citations; AC, average citations; TLS, total link strength.

### 3.3 Analysis of authors and co-cited authors

**Figure 4:**
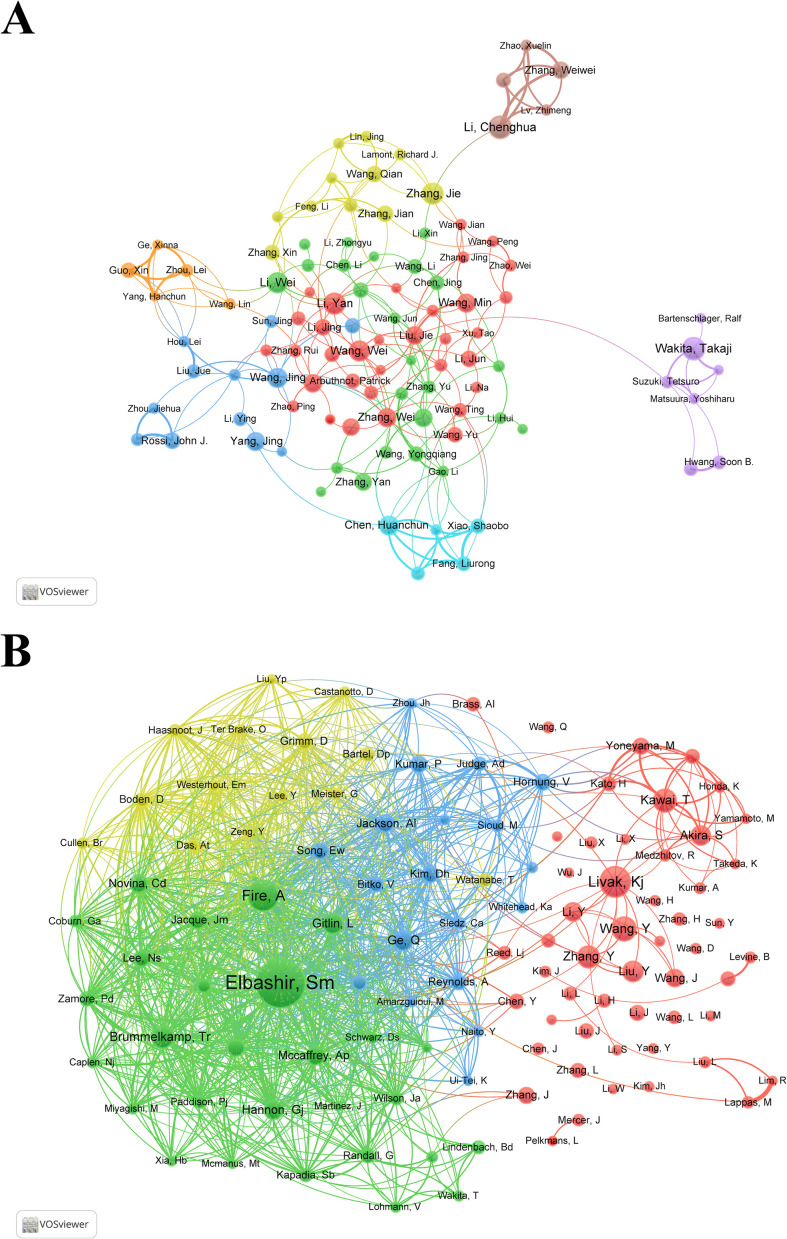
**(A)** Collaborative network visualization and cluster analysis of authors. **(B)** Collaborative network visualization and cluster analysis of co-cited authors.

Representative scholars and research teams in the field of siRNA therapeutics for microbial infections can be identified through author and co-cited author analysis. **Table 3** lists the top 10 most prolific authors, each having published over 20 articles. Wakita, Takaji and Li, Chenghua tied for first place with 25 articles. **Figure 4A** illustrates the collaborative network of authors with 10 or more publications, highlighting the active scholars with extensive collaboration in this field. **Supplementary Table S2** and **Figure 4B** provide a statistical and clustering analysis of co-cited authors. Among the top 10 co-cited authors, Elbashir, SM, Fire, A, and Livak, KJ ranked the highest in co-citation, while Elbashir, SM, Fire, A, and Brummelkamp, TR led in TLS, indicating their pivotal roles as pioneers and significant contributors to the field.

**Table 3:** Top 10 highly productive authors.

Rank	Author	Country	PN	TC	AC	TLS
1	Wakita, Takaji	Japan	25	1647	65.88	82
2	Li, Chenghua	China	25	335	13.40	70
3	Zhang, Jie	China	23	504	21.91	68
4	Li, Yan	China	23	465	20.22	25
5	Lappas, Martha	Australia	23	386	16.78	29
6	Li, Wei	China	22	618	28.09	31
7	Wang, Wei	China	22	586	26.64	36
8	Kohl, Alain	England	21	891	42.43	75
9	Chen, Huanchun	China	21	679	32.33	83
10	Wang, Jing	China	21	379	18.05	52

PN, publications number; TC, total citations; AC, average citations; TLS, total link strength.

### 3.4 Analysis of journal and co-cited journal

**Figure 5:**
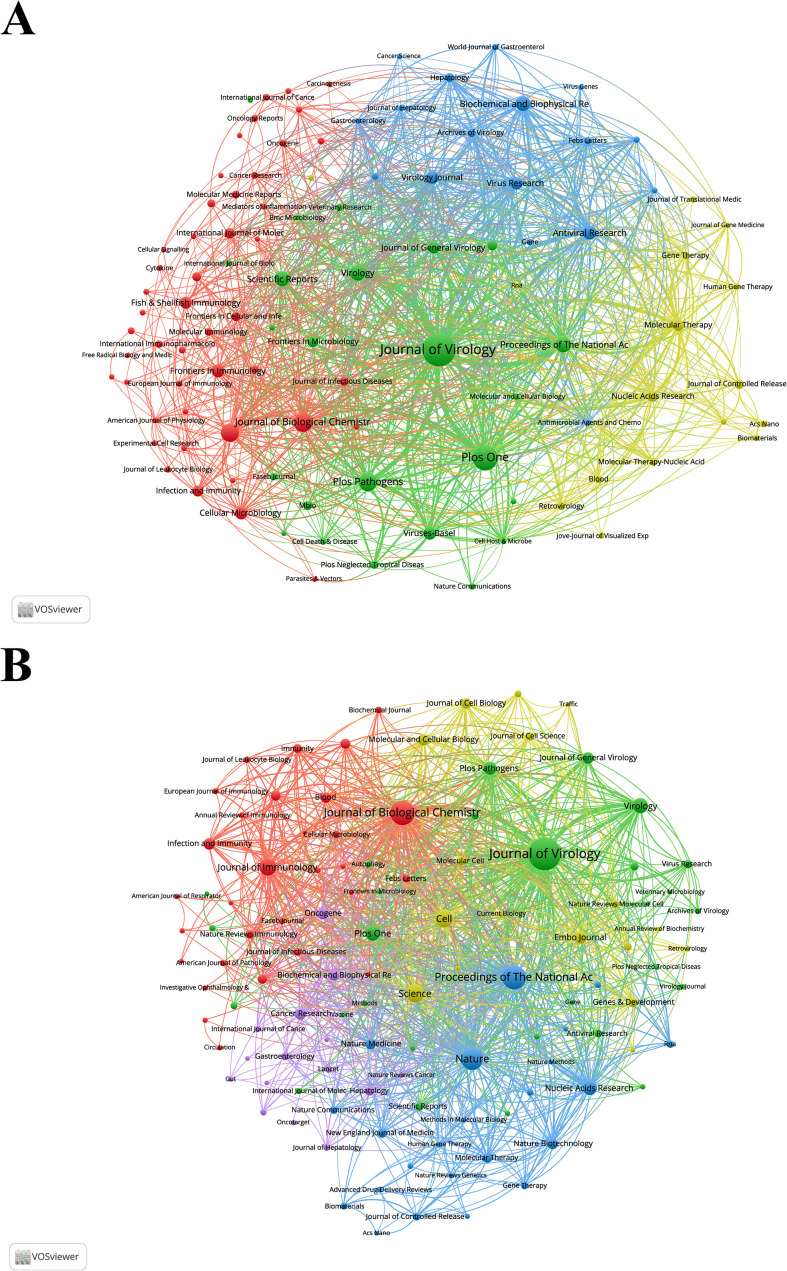
**(A)** Collaborative network visualization and cluster analysis of journals. **(B)** Collaborative network visualization and cluster analysis of co-cited journals.

Since 2001, research on siRNA therapeutics for microbial infections has been published in 1,234 academic journals. **Table 4** and **Supplementary Table S3** list the top 10 most productive and co-cited journals. Journals such as Journal of Virology, Journal of Biological Chemistry, Journal of Immunology, Proceedings of the National Academy of Sciences of the United States of America, PLOS One, and Virology appear in the top 10 of both lists, signifying their central role in this research area. The Journal of Virology stands out as the most productive and influential journal. The collaborative network of journals is presented in **Figure 5A**, while **Figure 5B** shows the co-citation network of journals.

**Table 4:** Top 10 highly productive journals.

Rank	Journal	Country	IF^*^	Quartile^*^	PN	TC	AC	TLS
1	Journal of Virology	USA	3.8	Q2	568	31650	55.72	2184
2	Plos One	USA	2.6	Q2	328	10253	31.26	779
3	Journal of Biological Chemistry	USA	3.9	Q2	189	11773	62.29	360
4	Plos Pathogens	USA	4.9	Q1	178	10536	59.19	567
5	Journal of Immunology	USA	3.4	Q2	177	11951	67.52	326
6	Virology	USA	2.4	Q3	134	5324	39.73	572
7	Proceedings of the National Academy of Sciences of the United States of America	USA	9.1	Q1	132	17852	135.24	972
8	Biochemical and Biophysical Research Communications	USA	2.2	Q3	126	2844	22.57	316
9	Scientific Reports	USA	3.9	Q1	125	2944	23.55	240
10	Antiviral Research	Netherlands	4.0	Q1	107	2849	26.63	761

*Reference to JCR 2024.

IF, impact factor; PN, publications number; TC, total citations; AC, average citations; TLS, total link strength.

### 3.5 Cluster and burst analysis of keywords

**Figure 6:**
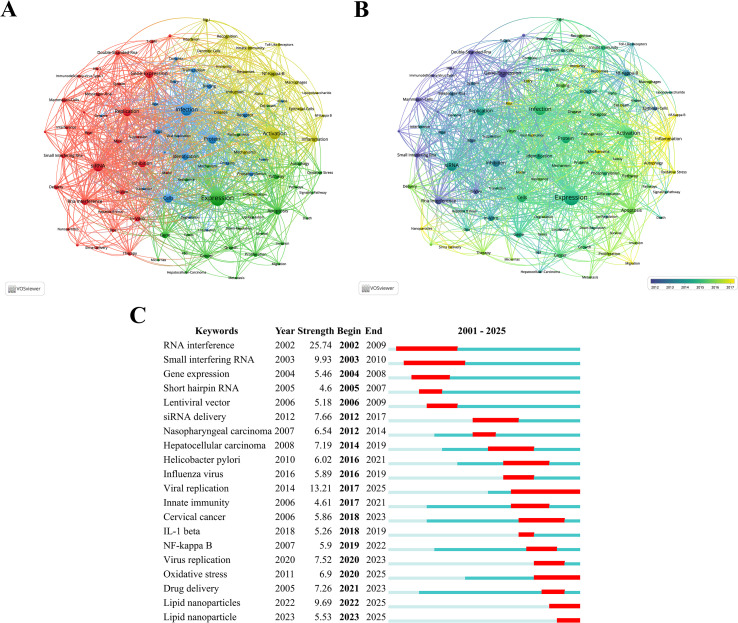
**(A)** Collaborative network visualization and cluster analysis of keywords. **(B)** Visualization of overlay time evolution for keywords. **(C)** The top 20 keywords with the most significant citation bursts, with red lines indicating the burst duration.

Keyword clustering is an effective method for identifying core topics and emerging trends in the field of siRNA therapeutics for microbial infections by analyzing co-occurrence relationships among keywords in the literature, thereby reflecting the dynamics of disciplinary development. Through VOSviewer analysis, 97 keywords with more than 100 occurrences were identified, forming four distinct clusters, as shown in **Figure 6A**. Cluster 1 (red) consists of 30 terms such as “siRNA,” “replication,” “gene expression,” “inhibition,” “RNA interference,” “*in-vitro*,” “double-stranded RNA,” and “mammalian cells,” focusing on the fundamental mechanisms of siRNA through RNAi. Cluster 2 (green) includes 26 terms like “expression,” “apoptosis,” “gene,” “pathway,” “cancer,” “proliferation,” “growth,” “autophagy,” and “oxidative stress,” highlighting research directions related to disease mechanisms and the development of targeted therapies. Cluster 3 (blue) encompasses 21 terms, such as “infection,” “protein,” “cells,” “identification,” “virus,” “receptor,” and “binding,” addressing antiviral strategies and host-virus interaction mechanisms. Cluster 4 (yellow) contains 20 terms, including “activation,” “NF-kappa-B,” “inflammation,” “induction,” and “innate immunity,” emphasizing inflammatory signaling pathways related to siRNA and their interaction with the immune system. **Figure 6B** provides further insights into the temporal evolution of these keywords.

The phenomenon of keyword citation bursts often signals the emergence of new research hotspots. **Figure 6C** displays the top 20 keywords with the most significant citation bursts identified by CiteSpace analysis, with red lines indicating the burst duration. Research hotspots can be categorized into three phases: the early phase (2001–2009) focused on exploring fundamental mechanisms, with burst keywords such as “RNA interference,” “small interfering RNA,” “gene expression,” “short hairpin RNA,” and “lentiviral vector.” The middle phase (2010–2018) witnessed a peak in publication output, accompanied by burst keywords like “siRNA delivery,” “nasopharyngeal carcinoma,” “hepatocellular carcinoma,” “Helicobacter pylori,” “influenza virus,” “viral replication,” “innate immunity,” “cervical cancer,” and “IL-1β.” The latest phase (2019–present) includes keywords like “NF-kappa B,” “virus replication,” “oxidative stress,” “drug delivery,” and “lipid nanoparticles.” Keywords such as RNAi, small interfering RNA, siRNA delivery, hepatocellular carcinoma, Helicobacter pylori, viral replication, cervical cancer, and oxidative stress have exhibited burst durations exceeding five years, indicating sustained research attention.

### 3.6 Citation and burst analysis of studies

**Figure 7:**
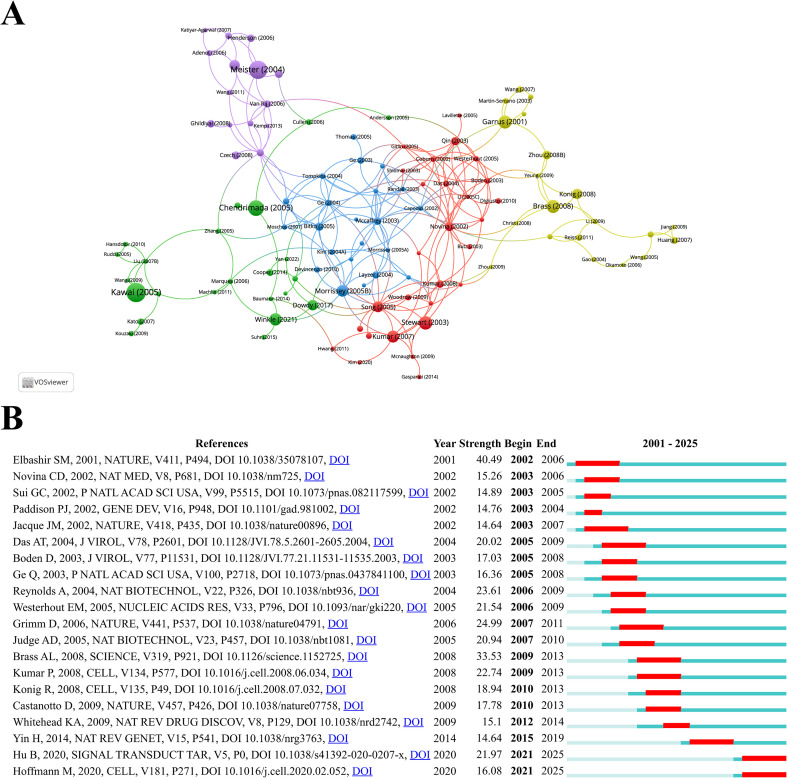
**(A)** Collaborative network visualization and cluster analysis of highly cited studies. **(B)** The top 20 references with the strongest citation bursts, with red lines indicating the burst duration.

Through citation analysis of the studies, representative articles in the field of siRNA therapeutics for microbial infections were identified. **Table 5** lists the top 10 most highly cited studies. The highest-ranked work is Taro Kawai’s 2005 study in Nature Immunology, titled “IPS-1, an adaptor triggering RIG-I- and Mda5-mediated type I interferon induction,” which demonstrated that siRNA-mediated “knockdown” of interferon-beta promoter stimulator 1 (IPS-1) could block virus-induced interferon activation. This study directly validated siRNA-guided interference effects and emphasized its potential as a gene-silencing tool. The second most cited study is Gunter Meister’s 2004 Nature paper, “Mechanisms of gene silencing by double-stranded RNA,” which elucidated the RNAi mechanism by revealing how double-stranded RNA (dsRNA) is processed into short RNA duplexes with distinct size and structure, while establishing their unique gene-silencing capabilities. Ranking third is Thimmaiah P. Chendrimada’s 2005 Nature study, “TRBP recruits the Dicer complex to AGO2 for microRNA processing and gene silencing,” which uncovered that the human immunodeficiency virus transactivating response RNA-binding protein (TRBP) regulates RISC assembly through interactions with Dicer and Argonaute 2. siRNA experiments confirmed its essential role in miRNA biogenesis and RNAi functionality. **Figure 7A** presents the collaboration networks of these highly cited studies.

**Table 5:** Top 10 highly cited studies.

Rank	First author	Title	Journals	Year	DOI	TC
1	Kawai, Taro	IPS-1, an adaptor triggering RIG-I- and Mda5-mediated type I interferon induction	Nature immunology	2005	10.1038/ni1243	2122
2	Meister, Gunter	Mechanisms of gene silencing by double-stranded RNA	Nature	2004	10.1038/nature02873	1948
3	Chendrimada, Thimmaiah P.	TRBP recruits the Dicer complex to Ago2 for microRNA processing and gene silencing	Nature	2005	10.1038/nature03868	1576
4	Stewart, Sheila A.	Lentivirus-delivered stable gene silencing by RNAi in primary cells	RNA	2003	10.1261/rna.2192803	1205
5	Garrus, Jennifer E.	Tsg101 and the vacuolar protein sorting pathway are essential for HIV-1 budding	Cell	2001	10.1016/s0092-8674(01)00506-2	1162
6	Brass, Abraham L.	Identification of host proteins required for HIV infection through a functional genomic screen	Science	2008	10.1126/science.1152725	1161
7	Winkle, Melanie	Noncoding RNA therapeutics - challenges and potential solutions	Nature reviews Drug discovery	2021	10.1038/s41573-021-00219-z	1067
8	Kumar, Priti	Transvascular delivery of small interfering RNA to the central nervous system	Nature	2007	10.1038/nature05901	1049
9	Morrissey, David V.	Potent and persistent *in vivo* anti-HBV activity of chemically modified siRNAs	Nature biotechnology	2005	10.1038/nbt1122	968
10	Song, Erwei	Antibody mediated *in vivo* delivery of small interfering RNAs via cell-surface receptors	Nature biotechnology	2005	10.1038/nbt1101	864

DOI, digital object unique identifier; TC, total citations.

CiteSpace analysis identified the top 20 references with the strongest citation bursts, visualized in **Figure 7B**, with burst durations marked in red. The first significant citation burst emerged in 2002, triggered by Elbashir SM’s landmark 2001 Nature study, “Duplexes of 21-nucleotide RNAs mediate RNAi in cultured mammalian cells,” which maintained the burst condition until 2006. This groundbreaking work experimentally demonstrated that 21-nucleotide siRNA duplexes could specifically inhibit endogenous and exogenous gene expression across multiple mammalian cell lines, highlighting their potential as functional genomics tools and gene-specific therapeutics. Notably, two recent burst studies include Bo Hu’s 2020 Signal Transduction and Targeted Therapy review, “Therapeutic siRNA: state of the art,” which synthesizes advancements in siRNA chemical modifications and delivery platforms, discussing both milestones and ongoing challenges in specificity and delivery. Another is Markus Hoffmann’s 2020 Cell paper, “SARS-CoV-2 Cell Entry Depends on ACE2 and TMPRSS2 and Is Blocked by a Clinically Proven Protease Inhibitor.” While not directly related to siRNA, its revelation of the ACE2/TMPRSS2 signaling pathway as critical for SARS-CoV-2 entry has been extensively cited in siRNA-based antiviral studies targeting this pathway.

## 4 Discussion

### 4.1 Global research status and trends

Over the past two decades, the field of siRNA therapeutics for microbial infections has experienced transformative growth, marked by evolving publication trends, shifting geographic contributions, and changing research priorities. A comprehensive bibliometric analysis of 8,426 publications in this study reveals a three-phase developmental trajectory: the early phase (2001–2009) was characterized by explosive growth, with a 110.53% annual increase in publication volume, focusing on RNAi mechanisms and validation; the middle phase (2010–2018) saw a period of dual-peak stabilization with growth rates below 1%, emphasizing drug delivery systems and clinical applications; the latest phase (2019–present) exhibits cyclical fluctuations and moderate declines in publication numbers, with a focus on COVID-19 pandemic-responsive innovations and novel strategies for siRNA therapeutics in specific infectious diseases. This progress has been driven by continuous technological advancements, including the elucidation of RNAi mechanisms, innovations in drug manufacturing, the development of novel delivery systems, and the emergence of combination therapeutic strategies, coupled with the urgency of global health challenges, particularly during the COVID-19 pandemic, which significantly accelerated the clinical translation of siRNA therapies.

Geographically, research output is primarily led by China and the USA, contributing 2,849 and 2,820 publications, respectively, accounting for 33.8% and 33.5% of the total publications. While China leads in publication volume, the USA maintains unparalleled academic influence, as evidenced by its higher total and average citation counts and its dominance in international collaboration networks. The steady increase in China’s publication share reflects its increased research investment and growing application scale. Notably, while China’s institutional output is concentrated within the Chinese Academy of Sciences, top institutions in the USA such as Harvard University, the University of Pennsylvania, and MIT lead high-impact studies, underscoring their pivotal role in pioneering clinical applications and innovations. There are two main reasons why China leads in publications but a lower total citations compared to the USA. First, the USA is the pioneer in this field and holds significant academic influence. Second, China’s publications is predominantly concentrated in the latest phase, resulting in a citation time-lag bias where recent studies tend to be cited less frequently. Additionally, countries like Japan, South Korea, Canada, and European nations, particularly Germany, France, and the UK, show lower publication volumes in comparison to China and the USA, likely due to disparities in population size and research investment. However, these nations have also made significant contributions, collectively establishing a complementary global framework for siRNA therapeutics alongside China and the USA. At the author level, Wakita, Takaji (Japan Institute for Health Security) and Li, Chenghua (Ningbo University) are the most productive authors, each with 25 publications. Their main research areas focus on siRNA therapeutic targets for hepatitis viruses and innate immune signaling pathways, respectively. However, foundational influence lies with co-cited pioneers such as Fire A (Nobel laureate for RNAi discovery) (Fire et al., 1998) and Elbashir SM (seminal 2001 Nature paper on siRNA-mediated RNAi in mammalian cells) (Elbashir et al., 2001a), whose work forms the theoretical basis of siRNA therapeutics. At the journal level, Journal of Virology is the most productive journal, while Nature holds the highest citation count, serving as key platforms for disseminating research in this field.

### 4.2 Early phase (2001–2009): mechanism of RNA interference

The early phase laid the conceptual and technological foundation for siRNA therapeutics against microbial infections, marked by explosive growth in publications and the discovery of RNAi’s foundational mechanisms. RNAi is a natural cellular process that silences gene expression by targeting and degrading mRNA. The seminal discovery of RNAi by Fire A and Mello C in 1998 revolutionized our understanding of gene regulation in cells (Fire et al., 1998), paving the way for the development of RNA-based therapeutics. However, initial applications of siRNA encountered challenges due to interferon responses triggered by dsRNA of varying lengths, which resulted in nonspecific mRNA degradation. To overcome this, researchers identified that dsRNA fragments with two-nucleotide 3′ overhangs could effectively degrade sequence-specific mRNA without eliciting interferon production (Elbashir et al., 2001b; Schubert et al., 2005). These siRNA molecules, typically 21–23 nucleotides in length, are designed to complement target mRNA sequences, enabling precise gene silencing in mammalian cells (Zamore et al., 2000; Elbashir et al., 2001a; Sørensen et al., 2003). The siRNA molecules bind to mRNA, either blocking its translation into proteins or inducing its degradation, thereby modulating gene expression (Lipardi et al., 2001; Shyu et al., 2008; Prévost et al., 2011). Moreover, researchers developed methods to achieve targeted gene silencing through synthetic siRNA delivery. These siRNA molecules, designed to specifically silence pathogenic genes, enter cells and trigger enzymatic cascades that form the RISC (Rand et al., 2004; Schwarz et al., 2004). The Dicer enzyme processes dsRNA into siRNA duplexes, which consist of a passenger (sense) strand and a guide (antisense) strand. The siRNA is then loaded into the RISC complex. Within RISC, strand separation occurs: the guide strand, with a more stable 5′ end, is retained, while the passenger strand is cleaved by AGO2 nuclease and degraded (Chendrimada et al., 2005). The guide strand-RISC complex then scans cellular mRNA for complementary sequences, and upon target recognition, RISC induces site-specific mRNA cleavage, silencing gene expression (Valencia-Sanchez et al., 2006; Ameres et al., 2007). Notably, dsRNA exceeding 30 nucleotides can trigger innate immune responses in mammalian cells, inducing interferon production as a defense mechanism against viral dsRNA generated during replication. Consequently, therapeutic siRNA design must optimize length to minimize unintended immunogenicity and determine the minimal effective concentrations for gene silencing (Judge and MacLachlan, 2008). RNAi plays a pivotal role in gene regulation and innate antiviral immunity. As emerging therapeutic agents in molecular biology and gene regulation, siRNA-based drugs show significant promise. Moreover, siRNA has become a pivotal tool in functional genomics research, marking the onset of this phase (Dorsett and Tuschl, 2004; Van De Water et al., 2006).

### 4.3 Middle phase (2010–2018): drug delivery and clinical application

The middle phase marked the transition of siRNA therapeutics from mechanistic exploration to clinical application, characterized by a peak in publications and significant breakthroughs in drug delivery systems. SiRNA therapeutics show immense promise in treating microbial infections; however, their clinical translation hinges on the safety and efficiency of siRNA delivery systems, which must overcome challenges such as instability, immunogenicity, limited tissue penetration, and potential off-target effects (Cullis and Felgner, 2024). To effectively target pathogens or specific cells, siRNA must reach the infection site without being cleared or degraded prematurely. The safety and efficiency of delivery systems play a critical role in determining whether these therapeutics can engage their intended targets and maximize therapeutic efficacy (Vicentini et al., 2013). During this phase, substantial advancements in delivery technologies, including viral vectors, LNPs, tri-GalNAc conjugates, polymer carriers, and chemical modifications, provided new therapeutic options for microbial infections.

Viral vectors, capable of mediating efficient gene transduction and enabling long-term expression, were extensively explored for delivering genes encoding short hairpin RNA (Moffat et al., 2006; Meerbrey et al., 2011; Börner et al., 2013; Osório et al., 2014). These vectors have the potential for a single administration to achieve continuous, long-term siRNA production *in vivo*, a feature highly attractive for chronic infections requiring prolonged treatment, such as HIV (Chung et al., 2014; Spanevello et al., 2016; Swamy et al., 2016) and HBV (Li et al., 2009, 2016; Xia et al., 2013). However, their clinical application has been hindered by immunogenicity concerns, risks of insertional mutagenesis, and limited nucleic acid payload capacity (Zhang et al., 2023; Zhou et al., 2009), leading to the shift toward non-viral vectors as the dominant delivery strategy.

Among these, LNPs have emerged as the most successfully developed non-viral platform. LNPs use ionizable cationic lipids to encapsulate siRNA efficiently under acidic conditions, forming stable nanoparticles. Upon intravenous injection, their near-neutral surface charge at physiological pH minimizes nonspecific interactions and toxicity (Patil and Panyam, 2009; Buyens et al., 2012). Once inside the cells, LNPs undergo protonation in the acidic environments of endosomes or lysosomes, which triggers a charge reversal, facilitating the dissociation of siRNA from the LNPs. This enables efficient “endosomal escape” and the release of functional siRNA into the cytoplasm (Kolli et al., 2013). An additional advantage of LNPs is their natural liver-targeting property. After intravenous injection, LNPs are coated by apolipoproteins and selectively internalized by hepatocytes *via* low-density lipoprotein receptors (Akinc et al., 2010). This liver targeting feature makes LNPs particularly suitable for treating liver-enriched viral infections such as HBV and hepatitis C virus (HCV) (Cho et al., 2009; Mével et al., 2010; Wooddell et al., 2013; Sato et al., 2017). Currently, ARB-1740, an LNPs-delivered siRNA therapeutic targeting HBV genes, has entered clinical trials and demonstrated sustained reductions in HBsAg levels (Thi et al., 2019). For liver-tropic pathogens, tri-GalNAc conjugation has revolutionized targeted delivery by exploiting hepatocyte-specific asialoglycoprotein receptors (Nair et al., 2014, 2017), making it one of the most promising delivery systems in clinical applications. Subcutaneously administered GalNAc-siRNA conjugates enable efficient hepatic uptake without the need for complex carriers. This strategy simplifies manufacturing, supports patient-friendly dosing, and underpins HBV therapeutics (Zimmermann et al., 2017; Foster et al., 2018). Several GalNAc-siRNA therapies, such as ARC-520 (Yuen et al., 2020, 2022b), JNJ-3989 (Yuen et al., 2022a), bersacapavir (Yuen et al., 2023), and VIR-2218 (Yuen et al., 2024), have entered clinical trials, demonstrating durable suppression of all HBV antigens and offering hope for the functional cure of hepatitis B. Due to the liver-targeting properties of the two primary delivery vehicles, LNPs and tri-GalNAc conjugation, research on siRNA therapeutics for liver viral infections has progressed significantly ahead of studies targeting other organs or tissues. While LNPs and tri-GalNAc conjugates demonstrated clinical promise, their translational bottlenecks, including liver-centric biodistribution limiting broader organ applications and manufacturing scalability challenges, as well as uncertain long-term safety profile and high production costs, remained critical barriers.

Polymeric siRNA delivery systems also hold promise. Polymeric carriers form stable nanocomplexes by encapsulating siRNA, effectively protecting it and prolonging its circulation time *in vivo*. Through chemical modifications or incorporation of targeted ligands, these polymers exhibit enhanced biocompatibility and specificity (Takemoto and Nishiyama, 2017). Key advantages include stability and the ability to facilitate endosomal escape. Despite ongoing challenges related to immunogenicity and large-scale manufacturing, these systems have shown promising antiviral efficacy in infection models, including HIV and HBV (Weber et al., 2008, 2012; Wooddell et al., 2013). As a new generation of carriers, extracellular vesicles (EVs) are generating significant interest. EVs can carry various nucleic acid molecules, including siRNA, and offer advantages such as low immunogenicity, ease of body barrier penetration, and the potential for target modification through engineering (Jiang et al., 2017; O’Loughlin et al., 2017; Guo et al., 2025). However, their application in treating microbial infections still requires extensive research and optimization. Additionally, while chemical modification is not a delivery carrier itself, it plays a pivotal role in enhancing the inherent characteristics of siRNA. Appropriate chemical modifications can significantly improve siRNA stability, immune evasion, and assembly efficiency with RISC. Existing studies have demonstrated that chemical modifications can enhance the antiviral efficacy of siRNA therapeutics (Marimani et al., 2013, 2015; Kalke et al., 2022).

### 4.4 Latest phase (2019–present): COVID-19 pandemic-driven innovation

The latest phase marked by the COVID-19 pandemic has driven innovation in siRNA therapeutics, characterized by a modest decline and cyclical fluctuations in publication numbers, as well as the integration of multiple therapeutic strategies. The pandemic has fundamentally transformed the trajectory of siRNA therapeutics, accelerating their clinical translation through unprecedented global collaboration and resource mobilization. To address SARS-CoV-2’s high mutability and respiratory tract tropism, siRNA therapeutics have advanced significantly across three areas. Firstly, in terms of delivery systems, researchers have developed novel LNPs for delivering anti-SARS-CoV-2 siRNA. One such innovation is stealth LNPs, which protect siRNA, enabling stable circulation in the serum while effectively targeting the lungs (Idris et al., 2021). Another breakthrough involves the DOTAP+MC3 LNPs, which reduce the proportion of cationic lipid 1,2-dioleoyl-3-trimethylammonium-propane (DOTAP) and introduce the ionizable lipid DLin-MC3-DMA (MC3). This modification reduces the positive charge of the delivery vector, minimizing immunogenicity and enabling targeted lung delivery after intravenous injection (Cheng et al., 2020; Idris et al., 2021). These advancements in delivery technologies have made LNPs a prominent research hotspot once again, highlighting their potential to target sites beyond the liver and lungs. Additionally, the development of new nanoscale polymers (Athaydes Seabra Ferreira et al., 2025) and EVs (Fu and Xiong, 2021) for SARS-CoV-2 has shown phased progress. Secondly, regarding target design, siRNA sequences have been tailored to target highly conserved regions of the SARS-CoV-2 genome, such as the RNA-dependent RNA polymerase (Xu et al., 2022) and nucleocapsid protein (N protein)-coding regions (Rabdano et al., 2024; Xue et al., 2024), with the aim of inhibiting multiple variants from Alpha to Omicron. An exciting development in this phase is the application of artificial intelligence (AI) to optimize target selection. A recent study leveraged generative AI models, DeepFrag and EMPIRE, to target the N protein and optimize the structure of phenanthridine SARS-CoV-2 inhibitors. These optimized compounds, with strong binding affinity, were subsequently validated for their antiviral activity *in vitro* (Xiang et al., 2024), demonstrating the promising role of AI in accelerating antiviral drug development and addressing viral mutations. Additionally, AI is being used to optimize siRNA delivery systems, including the prediction of LNP characteristics and formulation optimization (Amoako et al., 2025), as well as in the broader fight against infectious diseases (Wong et al., 2023). Thirdly, regarding administration routes, the first inhaled siRNA drug, SNS812, for treating SARS-CoV-2, has been developed and entered clinical trials (Chang et al., 2022, 2025). Inhaled drug delivery represents a major innovation in achieving targeted lung delivery, directly delivering siRNA to respiratory tract infections. This method increases drug concentration in the lungs while reducing systemic exposure, thereby minimizing side effects. This advancement marks a significant step in the development of siRNA therapeutics for respiratory infections. Furthermore, preliminary breakthroughs have been achieved in the delivery to major organs or targets such as brain, tumor, muscle, spleen, and kidneys (Khare et al., 2023; Song et al., 2024; Vaidya et al., 2024; Quijano et al., 2025), offering new potential for extrahepatic targeted therapies.

Currently, siRNA therapeutics have achieved the greatest success in antiviral applications within microbial infections, largely due to the RNAi mechanism’s ability to effectively suppress viral pathogenesis. Beyond its relatively established applications in challenging viral infections such as HBV, HIV, and SARS-CoV-2, siRNA-based approaches have also been explored to varying extents for treating infections caused by respiratory syncytial virus, influenza, herpesviruses, Ebola, Zika, enteroviruses, dengue virus, and rabies virus (Liang et al., 2015; Martin et al., 2018; Martín-Vicente et al., 2019; Scott and Nel, 2021; Kalke et al., 2022; Zhang et al., 2022; Haas et al., 2023; Bie et al., 2024). In contrast, siRNA therapeutics have encountered significant obstacles in antibacterial applications. Bacteria lack the core RNAi machinery components, specifically the RISC, rendering siRNA ineffective for directly regulating bacterial genes. Furthermore, the historical absence of efficient delivery systems for bacterial cells has hindered progress. However, the emergence of EVs, particularly exosomes, as a novel biological delivery system, has begun to address this limitation. A groundbreaking study demonstrated the use of exosomes to deliver siRNA-AGO2 complexes, successfully suppressing the expression of drug resistance genes in MRSA. This innovative technology significantly reduced resistance protein levels, restoring MRSA’s sensitivity to antibiotics and providing a transformative strategy against global superbug infections. Additionally, siRNA has been applied to silence genes associated with *Helicobacter pylori* cytotoxin production and urease enzyme activity, effectively reducing inflammatory responses and colonization rates, which aids in the treatment of gastric cancer (Motamedi et al., 2023; Reza et al., 2023). For fungal pathogens, which possess primitive RNAi mechanisms, siRNA could theoretically be designed to silence drug resistance or virulence genes directly. Studies have confirmed siRNA-mediated silencing of critical genes in pathogenic fungi such as *Aspergillus fumigatus* and *Mucor* species, although these results are confined to *in vitro* and animal models (Guo et al., 2012; Nicolás et al., 2015; Yu et al., 2025). Practical challenges remain, as exogenous siRNA exhibits lower silencing efficiency in fungal cells compared to mammalian cells (Sahay et al., 2013; Dong et al., 2019; Mosquera et al., 2025). Consequently, siRNA-based antifungal therapies are still in a much earlier exploratory phase compared to viral and bacterial applications.

### 4.5 Limitations

Although bibliometric analysis provides a comprehensive and objective overview, several limitations must be acknowledged. Firstly, this study relied solely on publications from the WOSCC database, which may not encompass all relevant research on siRNA therapeutics. To ensure the integrity of citation data, maintain data consistency, and literature quality, joint analyses utilizing multi-source databases such as PubMed and SCOPUS were excluded. Secondly, our exclusive focus on English-language literature may result in the exclusion of studies published in other languages, potentially introducing language bias. In addition, there is a time-lag bias in citation analysis. This means that compared with earlier studies, recent studies may receive fewer citations due to being newly published. Thirdly, keyword-based retrieval strategies, such as “small interfering RNA” or “siRNA”, may inadvertently exclude pioneering terminology from early studies or alternative terminology of emerging fields. This inherent limitation of bibliometric methods underscores the necessity for iterative keyword refinement in future analyses. Lastly, the quality of the literature was not evaluated, meaning that high-quality and low-quality studies were treated equally. These inherent limitations should be considered when interpreting the results.

## 5 Conclusion

In conclusion, siRNA therapeutics have emerged as a promising approach for treating a wide range of diseases, with clinical applications spanning cancer, genetic disorders, microbial infections, and inflammatory diseases. This study examines the evolving landscape of siRNA therapeutics for microbial infections over the past two decades through bibliometric analysis. The field has progressed from foundational mechanistic studies to clinical applications, fueled by technological innovations and the urgency created by the COVID-19 pandemic. Current research hotspots include delivery systems, target selection, manufacturing technologies, antiviral therapeutics, and combination strategies. Future research should focus on organ-specific delivery beyond the liver, explore diverse administration routes, integrate AI with drug design, and foster global collaborations. By addressing existing challenges, siRNA-based therapies hold significant potential for advancing the treatment of microbial infections and offering transformative solutions for the future.

## Data Availability

The original contributions presented in the study are included in the article/**Supplementary Material**. Further inquiries can be directed to the corresponding author/s.
