# Supplementary material for: Development of siRNA therapeutics to combat microbial infections: a bibliometric analysis
Source: Front Cell Infect Microbiol. 2025 Oct 24;15:1697880. doi: 10.3389/fcimb.2025.1697880 (PMC12592196; doi:10.3389/fcimb.2025.1697880)
Supplement: Supplementary file 1 [file Table1.docx]

Supplementary Material

# Supplementary Tables

Supplementary Table S1. Top 10 institutions in total citations.

| Rank | Institutions | Country | PN | TC | AC | TLS |
| --- | --- | --- | --- | --- | --- | --- |
| 1 | Harvard Univ | USA | 122 | 13816 | 113.25 | 113.25 |
| 2 | Univ Penn | USA | 65 | 7146 | 109.94 | 109.94 |
| 3 | MIT | USA | 43 | 6057 | 140.86 | 140.86 |
| 4 | Univ Calif Los Angeles | USA | 42 | 5824 | 138.67 | 138.67 |
| 5 | Osaka Univ | Japan | 80 | 5476 | 68.45 | 68.45 |
| 6 | Inst Pasteur | France | 75 | 5070 | 67.60 | 67.60 |
| 7 | Chinese Acad Sci | China | 141 | 4759 | 33.75 | 33.75 |
| 8 | Univ Calif San Diego | USA | 46 | 4667 | 101.46 | 101.46 |
| 9 | Univ Tokyo | Japan | 95 | 4516 | 47.54 | 47.54 |
| 10 | Univ Massachusetts | USA | 43 | 4486 | 104.33 | 104.33 |

Abbreviation: PN, publications number; TC, total citations; AC, average citations; TLS, total link strength; MIT: Massachusetts Institute of Technology.

Supplementary Table S2. Top 10 highly co-cited authors.

| Rank | Co-cited authors | Country | TC | TLS |
| --- | --- | --- | --- | --- |
| 1 | Elbashir, SM | Germany | 839 | 6847 |
| 2 | Fire, A | USA | 474 | 3698 |
| 3 | Livak, KJ | USA | 464 | 874 |
| 4 | Wang, Y | China | 344 | 650 |
| 5 | Kawai, T | Japan | 319 | 1020 |
| 6 | Brummelkamp, TR | Netherlands | 312 | 2859 |
| 7 | Zhang, Y | China | 306 | 731 |
| 8 | Gitlin, L | USA | 280 | 2607 |
| 9 | Liu, Y | China | 276 | 513 |
| 10 | Ge, Q | USA | 269 | 2073 |

Abbreviation: TC, total citations; TLS, total link strength.

Supplementary Table S3. Top 10 highly co-cited journals.

| Rank | Co-cited journals | Country | IF^*^ | Quartile^*^ | TC | TLS |
| --- | --- | --- | --- | --- | --- | --- |
| 1 | Journal of Virology | USA | 3.8 | Q2 | 25297 | 872931 |
| 2 | Journal of Biological Chemistry | USA | 3.9 | Q2 | 14352 | 491353 |
| 3 | Proceedings of the National Academy of Sciences of the United States of America | USA | 9.1 | Q1 | 13866 | 521875 |
| 4 | Nature | UK | 48.5 | Q1 | 11368 | 410157 |
| 5 | Cell | USA | 42.5 | Q1 | 7971 | 307545 |
| 6 | Science | USA | 45.8 | Q1 | 7923 | 304691 |
| 7 | Journal of Immunology | USA | 3.4 | Q2 | 7307 | 234086 |
| 8 | Virology | USA | 2.4 | Q3 | 5831 | 235002 |
| 9 | Plos One | USA | 2.6 | Q2 | 5340 | 178709 |
| 10 | Nucleic Acids Research | UK | 13.1 | Q1 | 4783 | 185693 |

* Reference to JCR 2024.

Abbreviation: IF, impact factor; PN, publications number; TC, total citations; AC, average citations; TLS, total link strength.
